# The genomics of sporadic and hereditary colorectal cancer

**DOI:** 10.1308/rcsann.2024.0024

**Published:** 2024-04-01

**Authors:** ID Sadien, RJ Davies, JMD Wheeler

**Affiliations:** ^1^ University of Cambridge, UK; ^2^ Cambridge University Hospitals NHS Foundation Trust, UK

**Keywords:** colorectal cancer, genomics, molecular subtypes, serrated pathway, microsatellite instability, chromosomal instability

## Abstract

Colorectal cancer (CRC) is a leading cause of cancer deaths worldwide. Over the past three decades, extensive efforts have sought to elucidate the genomic landscape of CRC. These studies reveal that CRC is highly heterogeneous at the molecular level, with different subtypes characterised by distinct somatic mutational profiles, epigenetic aberrations and transcriptomic signatures. This review summarises our current understanding of the genomic and epigenomic alterations implicated in CRC development and progression. Particular focus is given to how characterisation of CRC genomes is leading to more personalised approaches to diagnosis and treatment.

## Introduction

Despite significant improvements in outcomes, led primarily by a better understanding of its pathophysiology and increased focus on its early detection, colorectal cancer (CRC) remains a leading cause of cancer-related mortality worldwide.^1^ A worrying phenomenon has been its increasing incidence in younger patients, which poses challenges for screening and treatment.^2^ While most CRC cases are sporadic, hereditary syndromes have offered valuable insights into the molecular mechanisms involved, which we have only recently begun to exploit. Genetic and transcriptomic profiling studies have revealed three key pathways that lead to CRC development: chromosomal instability (CIN), microsatellite instability (MSI) and serrated neoplasia pathways. This review provides an up-to-date summary of the molecular biology of CRC aimed at a surgical audience.

## Cell of origin

**Figure 1:**
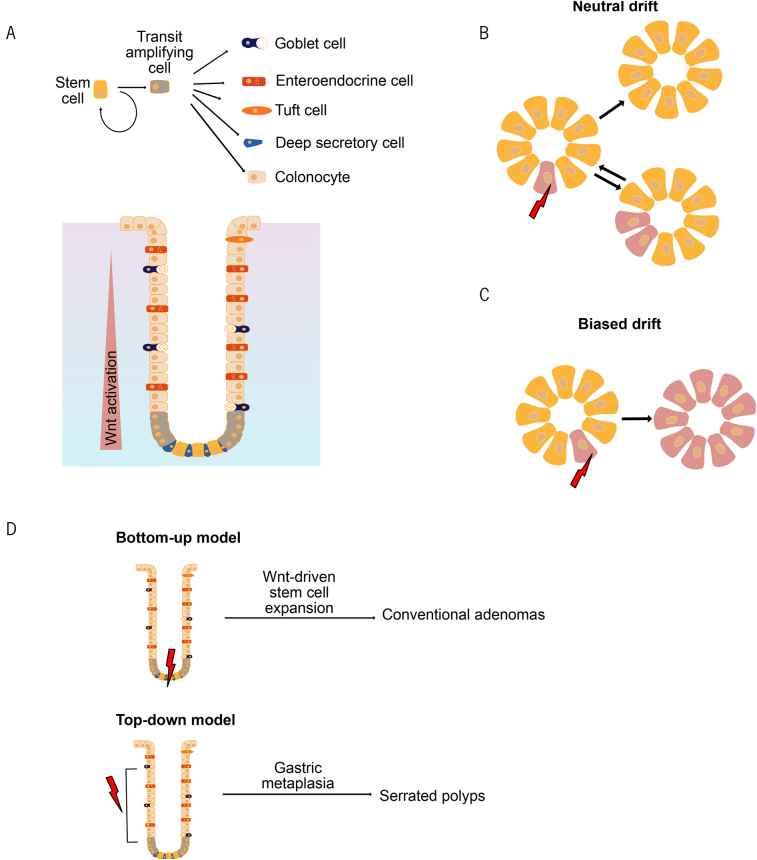
Stem cell dynamics and cancer initiation. (A) Cartoon of a longitudinal section of colonic crypt showing cellular hierarchy and organisation. (B) Cross-section of intestinal crypt showing neutral drift model. A stem cell with a neutral somatic mutation (red cell) will either get displaced from the stem cell compartment by a wildtype neighbour or divide to occupy more of the stem cell compartment with equal probability. (C) A stem cell with an advantageous mutation will populate the whole crypt, which is said to be monoclonally converted. (D) Schematic diagram showing the two proposed models of colorectal cancer initiation.

Rapid turnover of the intestinal epithelial lining is maintained by self-renewing pluripotent intestinal stem cells (ISCs), which are located at the bottom of intestinal crypts.^3^ ISCs give rise to transit-amplifying cells, which generate differentiated epithelial cells of absorptive and secretory lineages (Figure 1A). Mutations that confer a competitive advantage or non-mutagenic factors, such as diet or ageing, have been shown to accelerate the dominance of a specific ISC in the stem cell compartment of the crypt (biased drift), leading to all progeny in the crypt harbouring the same alteration (monoclonal conversion) (Figures 1B and 1C).^4^ Coupled with their long life and high Wnt activation, this has led to the belief that ISCs are the cells of origin of intestinal tumours.

This has been confirmed experimentally in mouse models, where targeted deletion of the *APC* gene in ISCs or activating mutations in *CTNNB1* (encoding β-catenin) lead to tumour formation originating from the bottom of the crypt (bottom-up model).^5^ However, the generalisability of this model has been questioned. For example, dysplastic areas (and *APC*-mutant regions) in sporadic adenomas have mostly been found on the luminal surface of the colon, with areas of normal crypt morphology underneath.^6^ A multiomics study published in 2021 has shed more light on this question by identifying two distinct transcriptional states for colorectal polyp formation, with one showing high levels of the classical stem cell marker *LGR5* in polyps with conventional adenoma histology, supporting a bottom-up model in this group, and the other displaying high levels of differentiation in polyps with serrated histology with gastric metaplasia, supporting a top-down model in this group of polyps (Figure 1D).^7^

## Chromosomal instability

**Figure 2:**
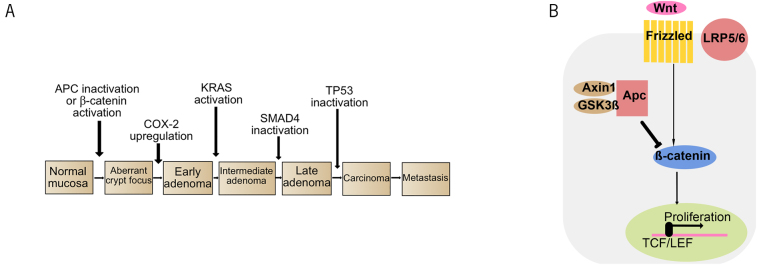
The conventional adenoma–carcinoma sequence begins with Wnt pathway activation. (A) The stepwise accumulation of mutations contributes to transformation and progression of colorectal cancer in the adenoma–carcinoma sequence. (B) Schematic diagram of the Wnt pathway.

Most CRCs follow the conventional adenoma–carcinoma sequence, which is characterised by the progressive accumulation of mutations in a number of well-recognised cancer driver genes (Figure 2A).^8^ This pathway begins typically with loss-of-function *APC* mutations. APC is a crucial regulator of the Wnt pathway, and plays a vital role in organogenesis and cellular proliferation.^9^ In the absence of Wnt ligands, APC forms a destruction complex with axin, casein kinase 1 and glycogen synthase kinase 3 beta, leading to the degradation of β-catenin (Figure 2B).^10^ However, following Wnt ligand activation, β-catenin translocates to the nucleus, where it binds to transcription factors of the TCF/LEF family, driving the expression of key genes involved in cell cycle regulation.^11^ Consequently, loss-of-function mutations in *APC* or (more rarely) gain-of-function mutations in *CTNNB1* result in overactivation of the Wnt pathway, leading to epithelial hyperproliferation and setting the stage for subsequent CRC development.

In the multistep model of colorectal carcinogenesis, these early Wnt pathway mutations are followed by mutations in *KRAS*, which activate pathways that promote cell proliferation and survival independent of epidermal growth factor receptor activation. The transition from adenoma to invasive cancer has been postulated to be associated with *TP53* mutations, which impair cell cycle arrest and DNA damage-induced apoptosis.^12^

Loss of APC function has also been implicated in CIN, which involves widespread copy number changes, structural variants and large-scale chromosomal rearrangements.^13^ Evidence for the role of APC in maintaining chromosomal integrity during mitosis comes from mouse experiments, in which *APC*-mutant embryonic stem cells have been shown to have extensive mitotic spindle aberrations.^14^

### Familial adenomatous polyposis syndrome

Familial adenomatous polyposis (FAP) syndrome is an autosomal dominant condition that predisposes affected individuals to CRC. Although it accounts for only 1% of CRC cases,^15^ the study of FAP has provided valuable insights into tumour formation in the colon. FAP occurs in approximately 1 in 8,300–14,000 individuals, with half of them developing colorectal adenomas by the age of 16 years.^16^ When left untreated, patients with FAP have a lifetime risk of CRC exceeding 90%.^17^ Individuals with FAP carry germline mutations in one allele of the *APC* gene. Interestingly, approximately 30% of patients with FAP have no family history and are therefore presumed to have *de novo* mutations.^18^

The phenotype of FAP (including the age of onset, type, and number of intestinal and extracolonic polyps) correlates with the specific type and location of *APC* mutations. Mutations located in the proximal portion of the APC protein (before codon 1,249) are associated with fewer than 1,000 polyps (sparse polyposis) whereas mutations between codons 1,250 and 1,330 are associated with more than 5,000 polyps (profuse polyposis). Mutations close to the N-terminus or distal to codon 1,578 result in a milder phenotype known as attenuated FAP, which is characterised by fewer than 100 polyps and a later age of onset.^19^ Roughly 10% of patients with FAP also develop intra-abdominal desmoid tumours.^20^

The diagnosis of FAP depends primarily on the degree of polyposis and a family history of colorectal adenomas. Individuals with 100 or more polyps, or a family history of FAP are clinically identified as having FAP. Confirmation of the diagnosis involves sequencing of the *APC* gene to identify a specific germline pathogenic variant. Owing to the risk of duodenal polyposis and cancer, patients with confirmed FAP are recommended to undergo screening gastroscopy starting at the age of 25 years and thereafter at regular intervals, guided by the Spigelman classification.^21^ Individuals with a first-degree relative diagnosed with FAP are considered at risk and are offered regular surveillance colonoscopies starting at 12–14 years of age.^22^

The management of FAP primarily involves surgical intervention. Prophylactic surgery is the preferred treatment option because of the high risk of CRC. Although non-steroidal anti-inflammatory drugs such as sulindac can induce polyp regression in some patients, the effect is not sustained after treatment cessation.^23^ Prophylactic surgery involves removing at-risk colorectal mucosa and restoring bowel continuity if feasible.

Surgical options include total colectomy with ileorectal anastomosis (IRA), proctocolectomy with ileal pouch–anal anastomosis (IPAA) or total proctocolectomy with end ileostomy.^19^ The advantages of IRA over IPAA (no routine requirement for a temporary defunctioning ileostomy, lower morbidity and better functional outcomes) need to be balanced with a risk of cancer formation in the rectal remnant.^24^ This decision is helped by a low pre-existing rectal polyp burden, and a patient with an understanding of the risks and who would be willing to undergo regular endoscopic surveillance. Additionally, patients with a germline *APC* mutation at codon 1,309 are known to be at increased risk of cancer in the rectal remnant.^25^

There is increasing recognition that the management of FAP (and other rarer gastrointestinal polyposis syndromes) should be undertaken in the context of specialised multidisciplinary teams. In the UK, a number of rare disease collaborative centres have been set up to standardise the care of these patients and accelerate research in these rare polyposis syndromes.^26^

## Microsatellite instability

**Figure 3:**
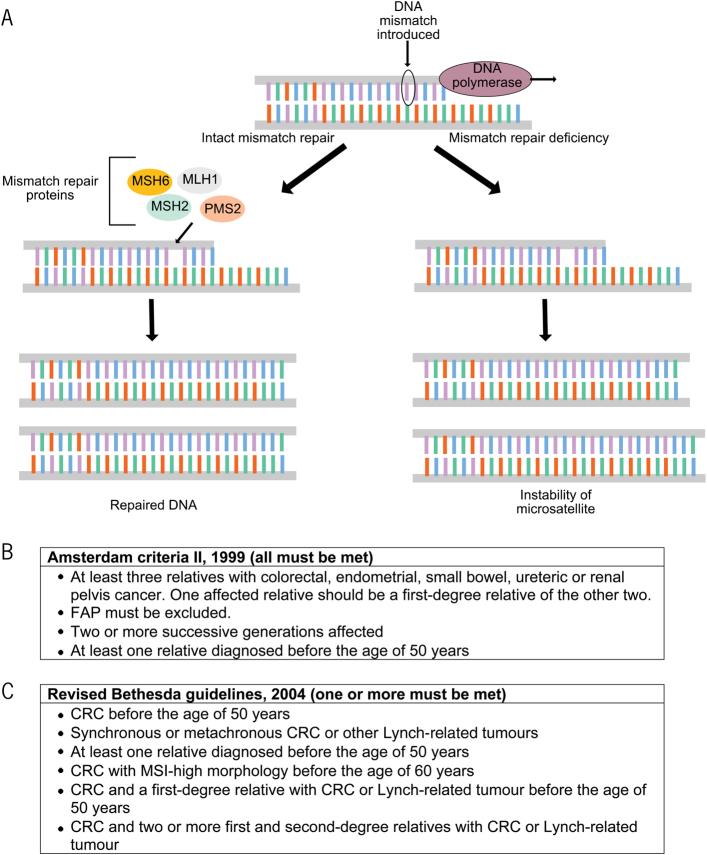
Mismatch repair deficiency leads to microsatellite instability. (A) DNA polymerase can lead to error-prone DNA replication, especially at microsatellites. Repair of these DNA mismatches by innate mechanisms maintains genomic stability (left). Defective mismatch repair leads to MSI (right). (B) Amsterdam criteria.^40^ (C) Revised Bethesda guidelines for patient selection for screening of Lynch syndrome.^41^ (A) is adapted from Eso *et al*,^27^ licensed under CC BY 4.0 (https://creativecommons.org/licenses/by/4.0/). CRC = colorectal cancer; FAP = familial adenomatous polyposis; MSI = microsatellite instability

MSI is a molecularly distinct pathway characterised by genetic alterations at the nucleotide level. Microsatellites (short repetitive sequences in genomic DNA) are prone to replication errors due to the slippage of DNA polymerases (Figure 3A).^27^ Although innate DNA repair mechanisms are very effective in correcting these errors, defects in these repair processes result in changes in these microsatellite sequences, leading to MSI and the accumulation of frameshift mutations in downstream genes.^28^

Approximately 15% of sporadic CRCs exhibit MSI due to somatic inactivation of DNA mismatch repair (MMR) genes.^29^ The most common MMR gene abnormality is hypermethylation of the *MLH1* promoter, leading to its downregulation. This is in contrast to hereditary forms of MSI, which are associated predominantly with germline mutations in *MLH1* and *MSH2*.^30^

Currently, two broad methods are available for the diagnosis of MMR deficiency and MSI in tumours. Immunohistochemistry to detect the expression of MMR proteins such as MLH1, MSH2, MSH6 and PMS2 in tumour tissue is perhaps the most widely used, and has excellent sensitivity (85–98%) and specificity (85–100%).^31,32^ Polymerase chain reaction-based methods are also commonly used to detect MSI by examining fragment lengths across a panel of loci that are commonly altered in these tumors.^33^

Accurate diagnosis of MSI has important clinical implications as MSI-high (MSI-H) CRCs have a better prognosis.^34^ This is thought to be due to the higher expression of tumour-specific peptides (neoantigens) that stimulate a strong anti-tumour immune response.^35^ Interestingly, these tumours also tend to be associated with a higher lymph node yield at resection, which partly explains the observation that resections with a higher lymph node yield are associated with improved survival.^36^

Additionally, MSI status is increasingly influencing treatment strategies, with immune checkpoint inhibitors showing effectiveness in MSI-H or deficient MMR (dMMR) CRCs. Indeed, a prospective phase 2 trial published in 2022 reported a complete clinical response in all cases of locally advanced dMMR rectal cancer treated with dostarlimab (an anti-PD-1 monoclonal antibody), thereby avoiding the need for chemoradiotherapy or surgery in this small cohort of patients.^37^ Immune checkpoint inhibition has also been shown to drastically increase the rate of pathological response in dMMR colon cancer while also reducing recurrence rates.^38^

### Lynch syndrome

Formerly known as hereditary non-polyposis colorectal cancer, Lynch syndrome is a cancer predisposition disorder associated with MSI. Lynch syndrome is thought to account for 1.7% of all CRC cases and 14.3% of cases in patients under the age of 50 years.^39^ Although it represents only a small proportion of all CRC cases, Lynch syndrome is associated with a significant risk of malignancy in the gastrointestinal tract, endometrium, ovaries and other organs. The underlying genetic basis involves germline mutations in DNA MMR genes, particularly *MLH1* or *MSH2*, which disrupt the intricate machinery responsible for maintaining genetic stability.

As individuals with Lynch syndrome face a strikingly elevated lifetime risk of CRC compared with the general population, surveillance colonoscopy plays a crucial role in the early detection and management of CRC in these patients.^22^ In order to aid in the identification of at-risk individuals, specific clinical criteria (such as the Amsterdam and Bethesda criteria) have been developed, incorporating family history and tumour characteristics (Figures 3B and 3C).^40,41^ Importantly, it has been estimated that 1 in 279 individuals carry mutations in MMR genes, with the vast majority being unaware of this.^42^ Consequently, cascade testing (i.e. informing relatives of a diagnosed individual and subsequently testing them) is crucial in guiding surveillance and offering prophylactic interventions.

Although its surgical management does not differ from that of sporadic CRC or FAP, a few recent studies have shown a beneficial role for aspirin in chemoprophylaxis to reduce the risk of CRC formation in patients with Lynch syndrome.^43^

## The serrated neoplasia pathway

The serrated neoplasia pathway has emerged as an important route for colorectal carcinogenesis, featuring serrated polyps as precursor lesions. This heterogeneous group of polyps exhibits a distinctive sawtooth infolding of the crypt epithelium.^44^ Approximately 15% of sporadic CRCs are believed to arise from the serrated pathway.^45^

The predominant molecular alterations in this pathway are *BRAF* (or more rarely, *KRAS*) mutations, particularly the *BRAF^V600E^* hotspot mutation or hypermethylation of CpG islands.^46^ *BRAF* mutations activate the MAPK/ERK pathway, and upregulate cellular proliferation and survival, thereby setting the stage for subsequent neoplastic transformation. On the left side of the colon, these mutations tend to lead to hyperplastic polyps or traditional serrated adenomas. Hypermethylation of CpG islands in tumour suppressor gene promoter regions leads to silencing of these genes. This tends to be more pronounced on the right side of the colon, leading to sessile serrated adenomas/polyps.

These tumours exhibit the CpG island methylation phenotype (CIMP-high).^47^ Although CpG island methylation is a physiological mechanism that regulates gene expression, it is unclear why this phenomenon is so pronounced in tumours arising from the serrated pathway. One hypothesis is that *BRAF* mutations (which tend to co-occur with CIMP) are involved.^48^ Occasionally, the MMR gene *MLH1* is affected, leading to MSI in a subset of these tumours.

Interestingly, CIMP and MSI are found at a much higher incidence in post-colonoscopy CRC, suggesting either that the serrated pathway is associated with accelerated carcinogenesis compared with the conventional adenoma–carcinoma pathway or that sessile lesions are more frequently missed during colonoscopy.^49^

### Serrated polyposis syndrome

Serrated polyposis syndrome (SPS) is the most common polyposis syndrome affecting the colon, with an estimated incidence of 1 in 100–200 screening colonoscopies.^50^ Although it is often grouped with other hereditary polyposes, no single germline alteration has been associated with the development of SPS. Germline mutations in *RNF43* have been reported in some patients with SPS but these mutations do not appear to be inherited in a simple Mendelian fashion.^51^ SPS is thought to confer a five-year CRC risk of 7% in patients who undergo regular surveillance.^52^

Diagnosis is based on the World Health Organization criteria:^53^

• Five or more serrated lesions/polyps proximal to the rectum, all being at least 5mm in size, with two or more being at least 10mm in size
• More than 20 serrated lesions/polyps of any size distributed throughout the large bowel, with at least 5 being proximal to the rectum

## Colitis-associated cancer

Chronic inflammation, particularly in individuals with inflammatory bowel disease, significantly increases the risk of CRC. Factors such as prolonged disease duration and inflammation intensity contribute to this heightened risk.^54^ The mechanisms underlying tumorigenesis during chronic inflammation are very likely multifactorial. Proinflammatory pathways involving signalling via NF-kB, IL-6/STAT3, COX2/PGE2 and IL-23/T_h_17 likely play a role in regulating the expression of inflammatory mediators and fostering a tumour-supportive microenvironment.^55^ Notably, the sequence of events in colitis-associated CRC differs from that of sporadic CRC, such as the early occurrence of *TP53* mutations detected not only in precancerous lesions but also in non-neoplastic inflamed mucosa. Moreover, colitis-associated CRC exhibits a lower prevalence of *APC* and *KRAS* mutations, suggesting a distinct pattern of mutation selection driven by chronic inflammation.^56^

## Transcriptomic classification of colorectal cancer

Efforts to functionally classify CRC based on mutations to aid prognostication and guide treatment decisions have been largely unsuccessful, with the notable exceptions of microsatellite/MMR status and a handful of actionable mutations in genes such as *BRAF* and *KRAS*. This is in contrast to transcriptomic classification, which has shown some promise.

**Figure 4:**
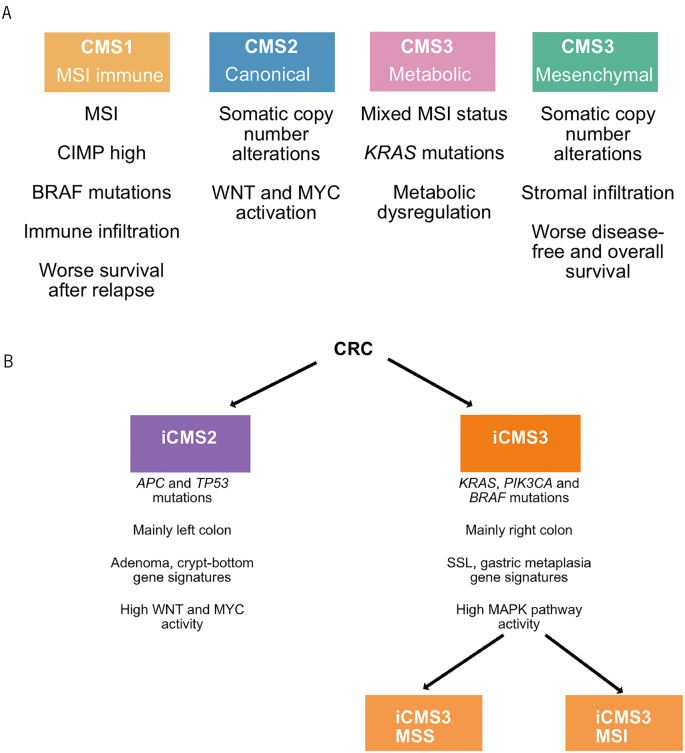
Classification of colorectal cancer based on transcriptional heterogeneity. (A) CMS classification derived from bulk RNA sequencing. (B) iCMS classification derived from single-cell and bulk RNA sequencing. Adapted from Joanito *et al*,^58^ licensed under CC BY 4.0 (https://creativecommons.org/licenses/by/4.0/). CMS = consensus molecular subtype; iCMS = intrinsic consensus molecular subtype; MSI = microsatellite instability; MSS = microsatellite stability; SSL = sessile serrated lesion

Consensus molecular subtype (CMS) classification was introduced to capture the molecular heterogeneity of CRC (Figure 4A).^57^ Four subtypes have been identified based on the differential gene expression: CMS1 is associated with MSI and *BRAF* mutations, CMS2 is characterised by Wnt and Myc activation and CIN, CMS3 is enriched for *KRAS* mutations and metabolic dysregulation, and CMS4 displays mesenchymal features with TGF*-*β pathway activation. Prognosis and treatment response vary considerably across subtypes, validating the value of transcriptomic classification. Nevertheless, CMS classification has limitations, such as being dominated by the stromal content of tumours, prompting the development of CRC intrinsic subtypes and intrinsic CMSs to overcome these challenges (Figure 4B).^58^

## Conclusions

Significant strides have been made over the past few decades in deepening our understanding of the genetic and molecular mechanisms underlying the development of CRC. The increasing accessibility and implementation of next-generation sequencing technologies in routine clinical practice has already begun to improve patient stratification and open up novel therapeutic options that have shown promise. As a direct corollary, there will be an increasing requirement for the CRC multidisciplinary team to be familiar with the results of these investigations and their implications for their patients.
